# ESA Hyporesponsiveness Is Associated with Adverse Events in Maintenance Hemodialysis (MHD) Patients, But Not with Iron Storage

**DOI:** 10.1371/journal.pone.0147328

**Published:** 2016-03-02

**Authors:** Takahiro Kuragano, Kenichiro Kitamura, Osamu Matsumura, Akihiko Matsuda, Taiga Hara, Hideyasu Kiyomoto, Toshiaki Murata, Shouichi Fujimoto, Hiroki Hase, Nobuhiko Joki, Atushi Fukatsu, Toru Inoue, Yukihiro Itakura, Takeshi Nakanishi

**Affiliations:** 1 Department of Internal Medicine, Division of Kidney and Dialysis, Hyogo College of Medicine, Nishinomiya, Japan; 2 The Third Department of Internal Medicine Faculty of Medicine, The University of Yamanashi, Chuo, Japan; 3 Department of Nephrology and Blood Purification, Saitama Medical Center, Saitama Medical University, Kawagoe, Japan; 4 Division of Nephrology and Dialysis, Department of Cardiorenal and Cerebrovascular Medicine, Faculty of Medicine, Kagawa University, Kida, Japan; 5 Division of Integrated Nephrology and Telemedicine, Tohoku Medical Megabank Organization, Tohoku University, Sendai, Japan; 6 Division of Nephrology and Rheumatology, Department of Internal Medicine, Faculty of Medicine, Fukuoka University, Fukuoka, Japan; 7 Department of Hemovascular Medicine and Artificial Organs, Faculty of Medicine, University of Miyazaki, Miyazaki, Japan; 8 Department of Nephrology, Toho University Ohashi Medical Center, Tokyo, Japan; 9 Department of Internal Medicine, Yachiyo Hospital, Anjyo, Japan; 10 Department of Internal Medicine, Higashikouri Hospital, Osaka, Japan; 11 Department of Internal Medicine, Itakura Clinic, Tokorozawa, Japan; Tokushima University Graduate School, JAPAN

## Abstract

**Objective:**

It has been reported that hyporesponsiveness to erythropoiesis-stimulating agent (ESA) is associated with adverse events in patients on maintenance hemodialysis (MHD). However, it has not been determined whether higher iron storage is associated with an improved response, including better survival, to ESA.

**Design and Method:**

We measured serum ferritin, hemoglobin (Hb), and transferrin saturation (TSAT) levels every three months for two years in 1,095 MHD patients. The weekly dose of ESA to Hb ratio was also calculated as an index of ESA responsiveness (ERI).

**Results:**

A significant correlation (p<0.001, R = 0.89) between ferritin and Hb was only observed in the patients with ferritin levels <50 ng/mL. High-dose (≥50 mg/week) intravenous iron administration, female sex, low serum albumin, and angiotensin-converting enzyme inhibitor/angiotensin receptor blocker use were significant predictors of a high ERI value (>280); however, serum ferritin and TSAT levels did not predict a higher ERI. In the time-dependent Cox hazard model, the risk for a composite event in the patients with a high ERI (≥280) and a high ferritin level (≥100 ng/mL) was significantly greater (hazard ratio [HR], 2.09, P = 0.033) than that for patients with a high ERI and a low ferritin (<100 ng/mL) level.

**Conclusion:**

Hb was dependent upon ferritin levels in patients with ferritin levels <50 ng/mL but not in patients with ferritin levels ≥50 ng/mL. Patients with hyporesponsiveness to ESA had a greater risk of composite events, but ERI was unrelated to iron storage.

## Introduction

Recently, several studies have demonstrated an association between hyporesponsiveness (i.e., resistance) to erythropoiesis-stimulating agents (ESA) and poor clinical outcomes, such as increased cardiovascular morbidity, faster progression to end stage kidney disease and all-cause mortality [1–4]. The definition of ESA hyporesponsiveness/resistance has been introduced to identify the inability to achieve or maintain target hemoglobin (Hb) levels despite higher than usual doses of ESA [5]. It has been long demonstrated that the factors affecting ESA hyporesponsiveness and the subsequent need for higher doses of ESA include iron deficiency, chronic hyperparathyroidism, low serum albumin, elevated aluminum level, malnutrition, suboptimal dialysis, and medications, such as angiotensin-converting enzyme inhibitors (ACE-I), angiotensin receptor blockers (ARB) and statins [6–7]. Among these factors, iron deficiency has been cited as the most common cause of hyporesponsiveness in maintenance hemodialysis (MHD) patients [6]. A previous study reported a significant relationship between iron indices (e.g., serum levels of ferritin and transferrin saturation [TSAT]) and the erythropoietin resistance index (ERI) [7]. A meta-analysis reported that patients on MHD respond better to ESA when they are treated with intravenous iron [8]. Furthermore, the Dialysis Patients’ Response to Intravenous Iron with Elevated Ferritin (DRIVE) study [9] reported that even in patients with high serum ferritin levels (>800 ng/mL), administration of ferric gluconate reduced the required ESA dose. These authors suggested that in the presence of hyporesponsiveness to ESA and repleted iron storage, a higher dose of intravenous iron could overcome functional iron deficiency. However, a recent systematic review and meta-analysis of randomized controlled trials revealed that although intravenous iron administration increased Hb levels and decreasing the need for red blood cell transfusions, intravenous iron administration could be associated with a significantly increased risk of infection (relative risk, 1.33; 95% confidence interval, 1.10 to 1.64) compared with oral iron supplementation or no iron supplementation [10]. In MHD patients with repleted iron storage, it has not been determined whether reducing the dose of ESA, along with a prolonged therapy of high dose intravenous iron therapy, is associated with better survival.

In the Prospective Study of Treatment for Renal Anemia on Prognosis in hemodialysis patients (TRAP) study, we recently demonstrated that a high risk of death and/or adverse events was associated with a consistently high ferritin level, large fluctuations in ferritin levels and a high dose of intravenous iron [11]. The distinguishing feature of this study is the observation that increased serum ferritin levels following iron administration could affect the prognoses of MHD patients with ferritin levels in the lower range of the KDIGO guidelines. In the present study, we performed a secondary analysis of the patients enrolled in the TRAP study in which we evaluated the relationships among ESA responsiveness, iron storage capacity, and adverse events in MHD patients.

## Materials and Methods

The design and methods of the TRAP study have been previously reported [11]. Briefly, the study design of TRAP study is a prospective, multi-center observational study, which has been conducted in Japan since June 2007. The aim of TRAP study is to evaluate the relationship between anemia treatment and the prognosis of MHD patients. Although observational period of original TRAP study is three years, this sub-analysis was conducted in two years. The protocol was approved in accordance with the ethical principles outlined in the 1975 Declaration of Helsinki by the Ethics Review Board of Hyogo College of Medicine (approval number: 419). Written informed consent was obtained from all patients. The study was registered with the University Hospital Medical Information Network (UMIN) Clinical Trial Registry (UMIN000000687).

### Patients

Patients who received MHD for under one year, patients over 75 years of age, patients with chronic inflammation, malignancies, hematological disorders, or severe liver dysfunction, and patients who received anti-inflammatory drugs or immunosuppressive agents were excluded from this study.

### Measurements

Hb, ferritin, TSAT, and albumin levels were measured every three months. The ESA and iron preparation doses were evaluated concurrently. The blood levels of β2 microglobulin (MG), creatinine (Cr), total protein (TP), albumin, total cholesterol (T-CHO), low-density lipoprotein cholesterol (LDL-CHO), high-density lipoprotein cholesterol (HDL-CHO), triglycerides (TG), calcium, phosphate, intact-parathyroid hormone (int-PTH), high-sensitivity C-reactive protein (hCRP), and prealbumin were measured every six months. The use of vitamin D or ACE-I/ARBs was confirmed every three months. The weekly dose of ESA to Hb ratio was calculated as an index of ESA responsiveness (ERI).

### Definition of each event

We defined ischemic heart disease (angina or acute myocardial infarction) and congestive heart failure as cardiovascular disease and cerebral infarction and defined cerebral hemorrhage as cerebrovascular disease. Angina, acute myocardial infarction, and congestive heart failure were diagnosed by electrocardiogram, blood biochemistry tests or cardiac ultrasound. Cerebral infarction and cerebral hemorrhage were diagnosed by computed tomography or magnetic resonance imaging.

### Statistical analysis

Determinants of a high ESA resistance index (ERI): In this study, the median ERI was 280. To analyze the determinants of a higher ERI (>280), we used a least absolute shrinkage and selection operator (LASSO) regularization analysis, which introduces an additional penalty, with a power raised on the weight vector [12]. The following independent variables were used: sex, age, time on dialysis, treatment time, etiology of kidney disease, body mass index (BMI), vitamin D use, ACE/ARB use, ERI, intravenous iron dose, and serum levels of ferritin, iron, TSAT, hCRP, β2-MG, Cr, TP, albumin, T-CHO, LDL-CHO, TG, calcium, phosphate, and int-PTH. Based on the lambda value (0.04701062) that minimized the mean squared error of cross-validation, we obtained a feature-selected model for each data set.

#### Comparison of clinical parameters among the ferritin groups

The Mann-Whitney U test was used to analyze the differences between the clinical parameters of the ferritin groups. Although there were few patients with ferritin levels >301 ng/mL compared with the other groups, we maintained the clinical significance without losing the power of analysis. Therefore, we categorized the patients into four groups according to their ferritin levels (<50, 51–100, 101–300, and >301 ng/mL). The potential association between Hb and ferritin was assessed by linear regression analysis, and P<0.05 indicated statistical significance.

#### Composite events and ESA dose or ERI

We defined the composite events as cerebro-CVD, infectious disease, hospitalization, and death. We compared the ESA dose or ERI at the time of the composite event with the ESA dose or ERI at the end of the observational period in the patients without composite events. Furthermore, we also compared the mean ESA dose or the ERI until the composite events occurred with the mean ESA dose or ERI in the patients without composite events during observational period. The Mann-Whitney U test was used to analyze the effect of ERI or ESA dose on the composite events.

#### Cut-off values of the ESA dose or ERI for the composite events

A receiver operating characteristic (ROC) analysis was used to determine the sensitivity and specificity of the ESA dose or ERI on the risk of developing composite events. The Hanley and McNeil [13] method was employed to compare the independent ROC curve data.

#### Determinants for the composite events

To examine the determinants of composite events, a LASSO analysis was employed using sex, age, time on dialysis, treatment time, etiology of kidney disease, history of CVD, BMI, vitamin D use, ACE-I/ARB use, ERI, intravenous iron dose, and serum levels of ferritin, iron, TSAT, hCRP, β2-MG, Cr, TP, albumin, T-CHO, LDL-CHO, TG, calcium, phosphate, and int-PTH as the independent variables. Based on the lambda value (0.0204112) that minimized the mean squared error from cross-validation, we obtained a feature-selected model for each data set. Previous studies have employed Cox's proportional hazard model for evaluating the relationship between clinical factors and adverse events or survivals. In this study, we used LASSO analysis for the following 2 reasons. 1) It is possible that among the clinical factors evaluated in this study, the mean levels until adverse events are more important than the levels at baseline and adverse events. Thus, we presumed that it is difficult to estimate the proportional hazard ratio for all clinical factors. 2) In this study, to evaluate the relationship between adverse events and clinical parameters, many clinical factors are needed as independent factors. However, compared to the number of clinical factors, the number of adverse events during the study period was low. It is possible for the estimated results, according to Cox's proportional hazard model, to become unstable.

#### Relationship between ERI or ferritin and composite events

The mean serum ferritin level of this study was 125.4±147.0 ng/mL, and the median was 79 ng/mL. Therefore, we set the cut off value for ferritin at 100 ng/mL. Moreover, the median ERI of this study was 280. We set the cut off level for ERI at 280. To evaluate the relationship between the ERI or ferritin levels and composite events, the patients were divided into 4 categories, according to their ERI and serum ferritin levels: ERI ≥280 and ferritin <100 ng/mL; ERI ≥280 and ferritin ≥100 ng/mL; ERI <280 and ferritin <100 ng/mL; and ERI <280 and ferritin ≥100 ng/mL. A time-dependent proportional hazards model was employed using each event as a dependent variable and the ERI and ferritin levels as independent variables. A patient’s sex, age, etiology, and serum levels of hCRP and albumin were used as covariates.

Statistical analyses were performed using SPSS ver.18.0 (IBM, Inc., Chicago, IL, USA), R ver.2.13.0 (R Core Team (2011). R: A language and environment for statistical computing. R Foundation for Statistical Computing, Vienna, Austria. URL http://www.R-project.org/) and MedCalc ver.12.7.7 (MedCalc Software, Ostend, Belgium).

## Results

### Patient characteristics

The mean age of 1,095 patients was 61.8 years, and 33.7% of patients had diabetes. The mean time on dialysis therapy was 106 months. The mean Hb and ferritin levels were 10.6±1.0 g/dL and 125.4±147.0 ng/mL, respectively. The mean ESA dose was 3,212±2,107 IU/week. The mean ERI was 308±219. The median dose of ESA was 3,164 IU/week, and the median ERI was 280. Oral iron treatment was used in 2.6% of the patients, while 20.8% of the patients were treated with intravenous iron (Table 1). Seventy-nine CVD cases, 368 cases of infection, 324 hospitalizations, and 36 deaths occurred during the study period. The 193 patients who received darbepoetin during the study period were excluded, depending on the protocol used.

**Table 1:** Characteristics of patients at baseline.

Number of patients	1,095	Hb(g/dL)	10.6±1.0
**Age (yo)**	**61.8 ± 9.9**	**Ferritin (ng/mL)**	**78.5(31.0, 164)**
**% of male**	**60.3**	**TSAT(%)**	**26.7±11.7**
**% of DM**	**33.7**	**Albumin (g/dL)**	**3.7±0.3**
**Time on dialysis (month)**	**106 ± 101**	**Pre-albumin (mg/dL)**	**33.3±7.9**
**BMI**	**21.7 ± 12.3**	**int-PTH (pg/mL)**	**124(62, 198)**
**Use of ACEI/ARB (%)**	**59.8**	**hCRP (mg/dL)**	**0.06(0.06, 0.2)**
**Use of vitamin D (%)**	**63.7**		
**Dose of ESA(IU/week)**	**3212 ± 2107**		
**ERI**	**308 ± 219**		
**% of ion administration**			
** Oral**	**2.6**		
** IV**	**20.8**		

Data are the mean ± SD.

Ferritin, hCRP, and int-PTH are expressed as median (25th-75th percentile).

BMI: body mass index, DM: diabetes mellitus, HD: hemodialysis, int-PTH: intact-parathyroid hormone, hCRP: high sensitive c reactive protein, Hb: hemoglobin, TSAT: transferrin saturation, ESA: erythropoiesis-stimulating agent, IV: intravenous.

### Serum ferritin categories

**Fig 1:**
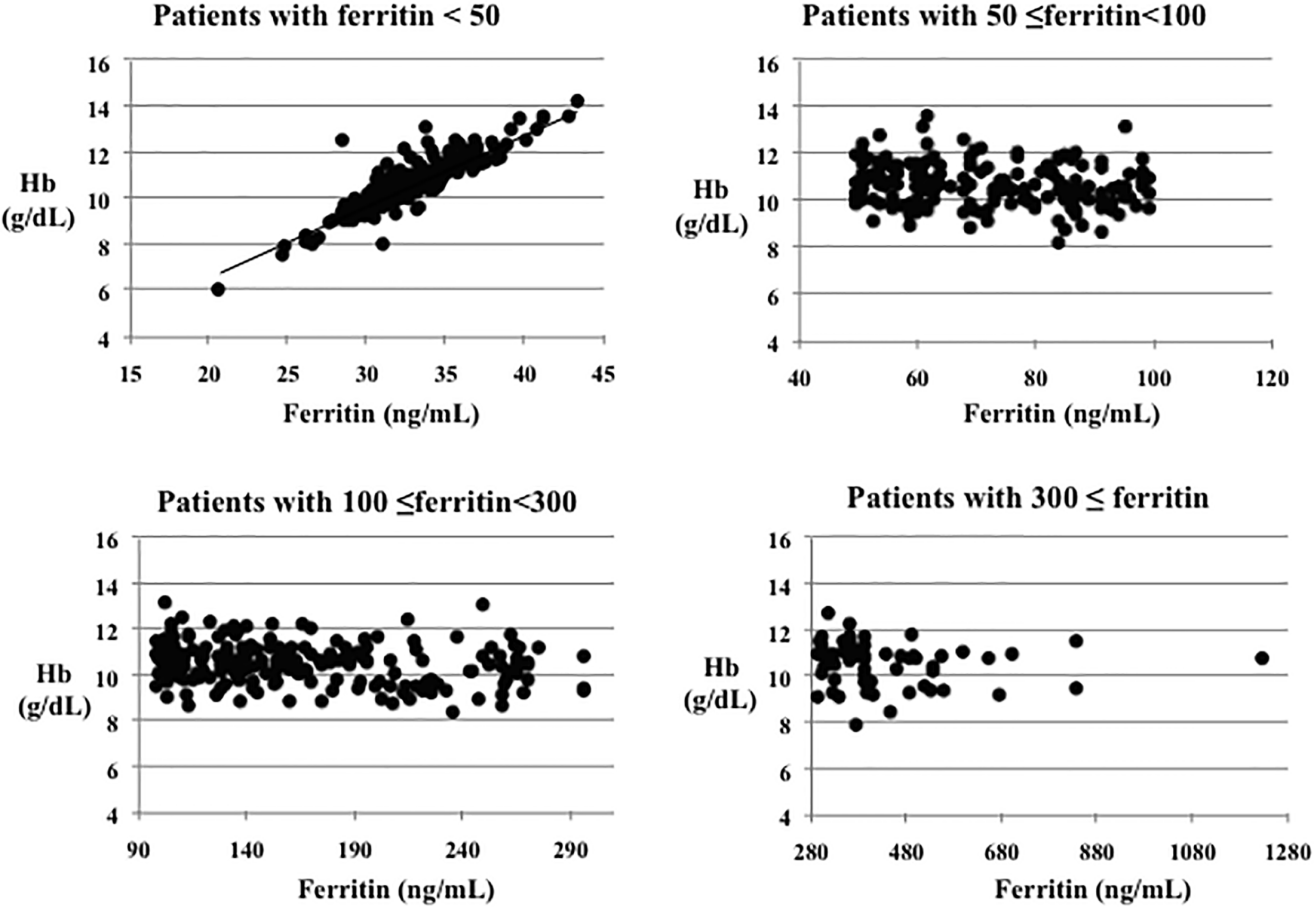
Relationship between Hb and ferritin levels among the patient groups, as stratified by serum ferritin levels. A significant correlation was found between Hb and ferritin levels only in patients with ferritin levels <50 ng/mL (p<0.001, R = 0.89). No significant correlation was found between Hb and ferritin levels in patients with 50–100, 100–300, and ≥300 ng/mL ferritin.

Patients were stratified into four groups, according to their serum ferritin levels (<50, 50–100, 101–300, and >300 ng/mL). There were no significant differences in Hb, albumin, prealbumin, and ERI levels, as well as ESA dose, between the groups. However, TSAT and hCRP levels were significantly lower (p<0.05) in the group with low serum ferritin levels (<50 ng/mL) compared to the groups with higher serum ferritin levels (101–300 or >300 ng/mL) (Table 2). A significant correlation (p<0.001, R = 0.89) between ferritin and Hb levels was observed only in the patients with ferritin levels <50 ng/mL. We found no significant relation between Hb and ferritin levels in the other groups (50–100, 100–300, or ≥300 ng/mL) (Fig 1).

**Table 2:** Anemia related factors among ferritin categories.

Ferritin categories	<50 (ng/mL)	51–100 (ng/mL)	101–300 (ng/mL)	>301 (ng/mL)
**Number of patients**	**341**	**228**	**255**	**78**
**% of male**	**63%**	**53%**	**56%**	**56%**
**Use of ACEI/ARB**	**28%**	**31%**	**30%**	**34%**
**Hb (g/dL)**	**10.62±1.04**	**10.61±0.92**	**10.45±0.88**	**10.45±0.93**
**Ferritin (ng/mL)**	**23(13.5, 34)**	72(60, 86.8)*	161.5(125, 213.3)*	400(334.5, 503.5)*
**TSAT (%)**	**23.71±9.41**	**25.62±9.71**	28.30±10.88*	33.00±16.75*
**Albumin (g/dL)**	**3.74±0.29**	**3.71±0.33**	**3.81±0.43**	**3.86±0.32**
**Pre-albumin (g/dL)**	**32.08±8.20**	**32.37±6.70**	**31.78±8.27**	**32.49±7.71**
**int-PTH (pg/mL)**	**131(63, 198)**	**126(61.5, 217)**	**121(59, 193)**	**120(72, 202)**
**hCRP (mg/dL)**	**0.06(0.02, 0.15)**	**0.06(0.02, 0.25)**	**0.06(0.03, 0.25)**	**0.12(0.04, 0.33)**
**Dose of ESA (IU/week)**	**3257.1±2034.2**	**3285.5±1834.9**	**3203.9±2152.6**	**3175.8±2157.8**
**ERI**	**320.4±223.1**	**315.2±189.0**	**310.4±217.9**	**315.7±234.0**

Data are the mean ± SD.

Ferritin, int-PTH, and hCRP are expressed as median (25th-75th percentile).

Hb: hemoglobin, TSAT: transferrin saturation, ESA: erythropoiesis-stimulating agent, hCRP: high sensitive c reactive protein.

*: compared with <50 ng/mL, P<0.05.

### Factors associated with ERI

**Fig 2:**
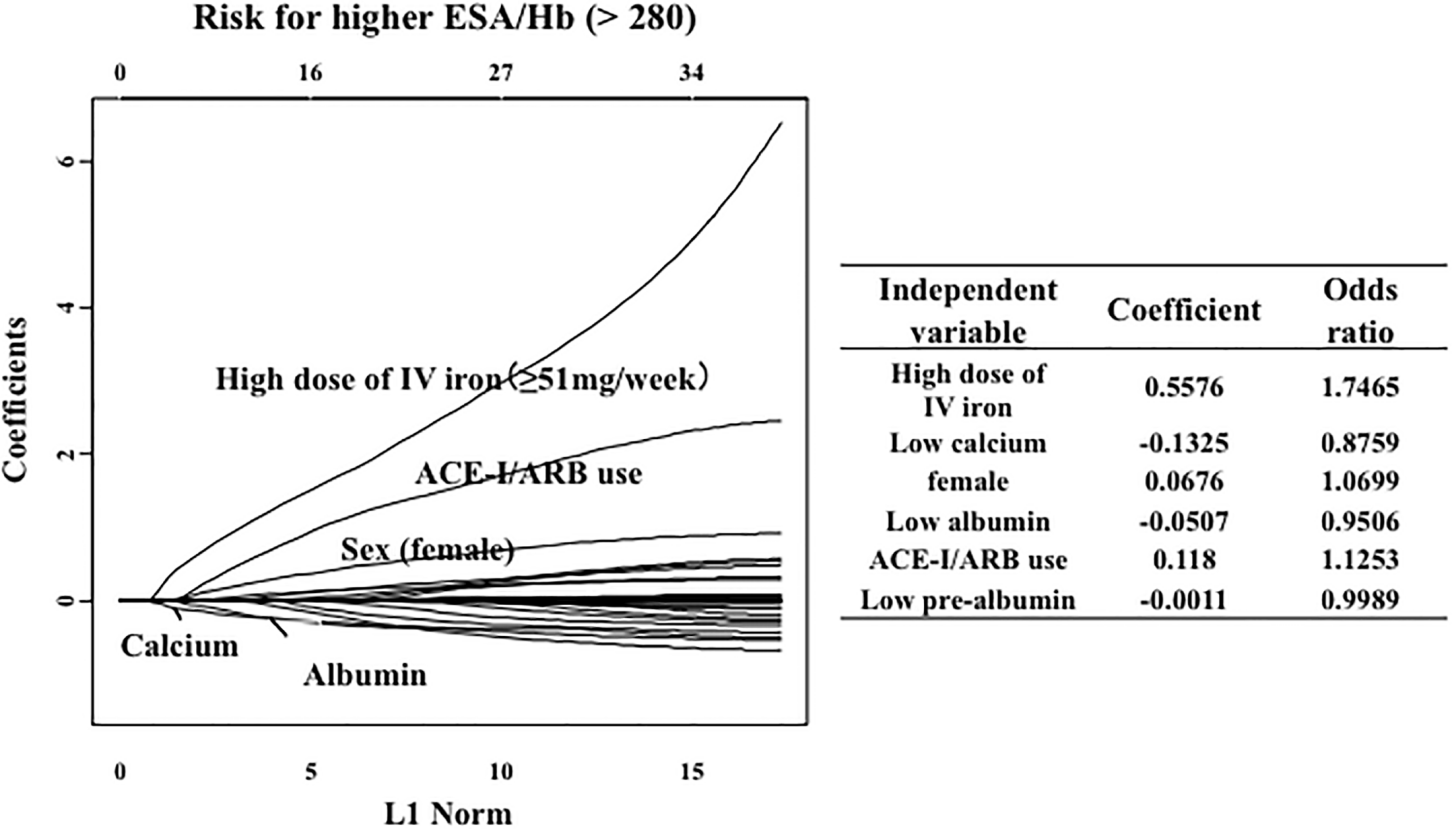
Factors associated with a high ERI (≥280). In the LASSO analysis, high doses of intravenous iron, low serum calcium levels, female sex, low serum albumin levels, angiotensin-converting enzyme inhibitor (ACE-I)/angiotensin receptor blocker (ARB) use, and low serum pre-albumin levels were selected significant predictors of high ERI scores.

Using LASSO regression analysis, a high dose (>50 mg/week) of intravenous iron administration (odds ratio, 1.746), a low serum calcium level (odds ratio, 0.8759), female gender (odds ratio, 1.0699), a low serum albumin level (odds ratio, 0.9506), ACE-I/ARB use (odds ratio, 1.1253) and a low serum prealbumin level (odd ratio, 0.9989) were selected as significant predictors of higher ERI (>280), whereas serum ferritin and TSAT levels were not significant predictors (Fig 2).

### Correlation among the ESA dose, ERI and composite events

**Fig 3:**
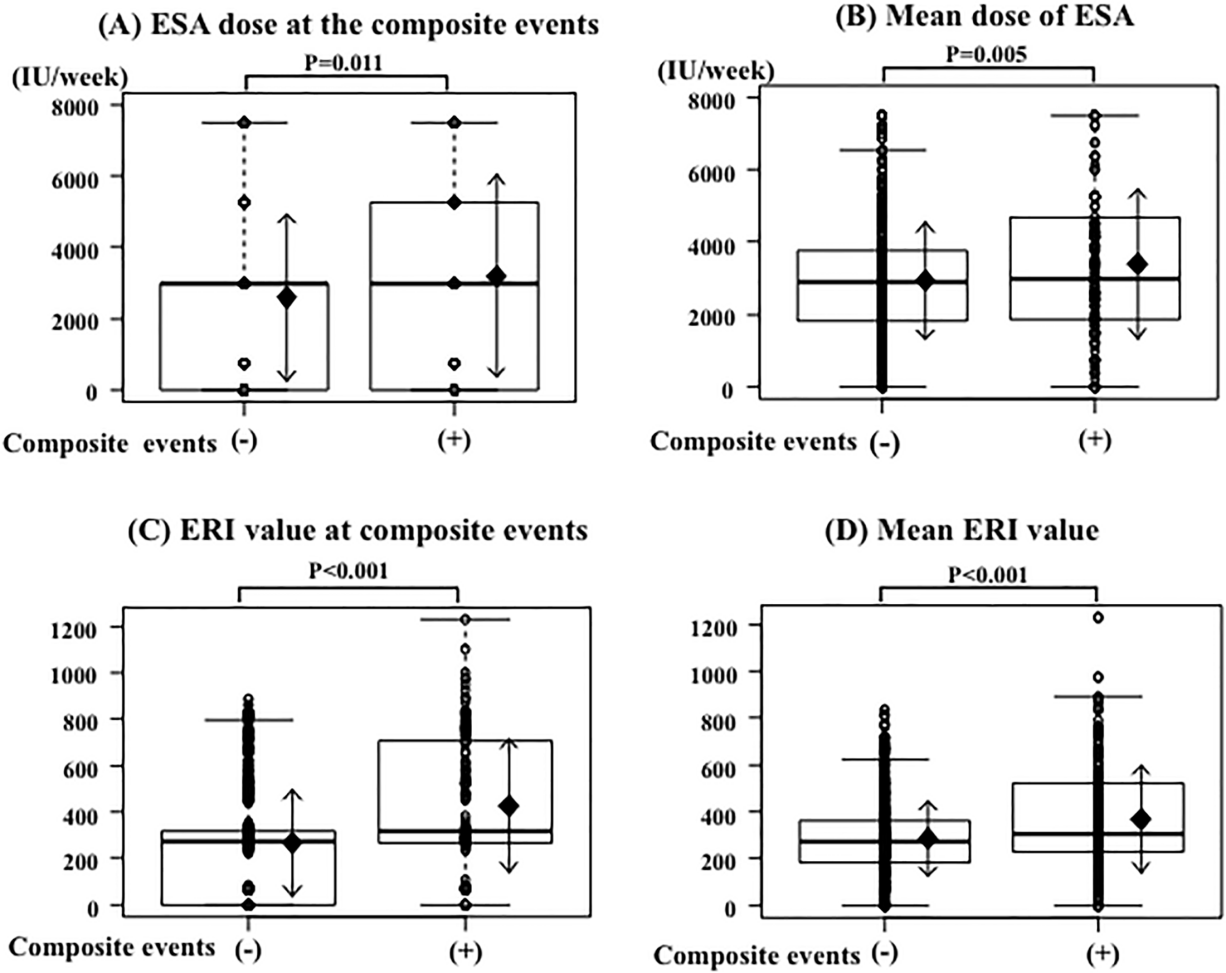
ESA dose and ERI in patients with or without composite events. (A) The ESA dose at the time of the composite event was significantly higher than that at the end of the observational period in patients without composite events. (B) The mean ESA dose (until the composite event) was significantly higher than the mean ESA dose during the observational period in patients without composite events. (C) The ERI at the time of the composite event was significantly higher than the ERI at the end of observational period in patients without composite events. (D) The mean ERI value (until the composite event) was significantly higher than the mean ERI value during the observational period in patients without composite events.

We analyzed the correlation between the composite events (CVD, infection, hospitalization, or death) and ESA dose or ERI using the Mann-Whitney U test. The ESA dose at the time of the composite events (p = 0.011) and the mean ESA dose in patients with composite events (p = 0.005) were significantly higher compared to those in patients without composite events. The ERI at the time of the composite events (p<0.001) and the mean ERI in patients with composite events (p<0.001) were also significantly higher than those in patients without composite events (Fig 3).

**Fig 4:**
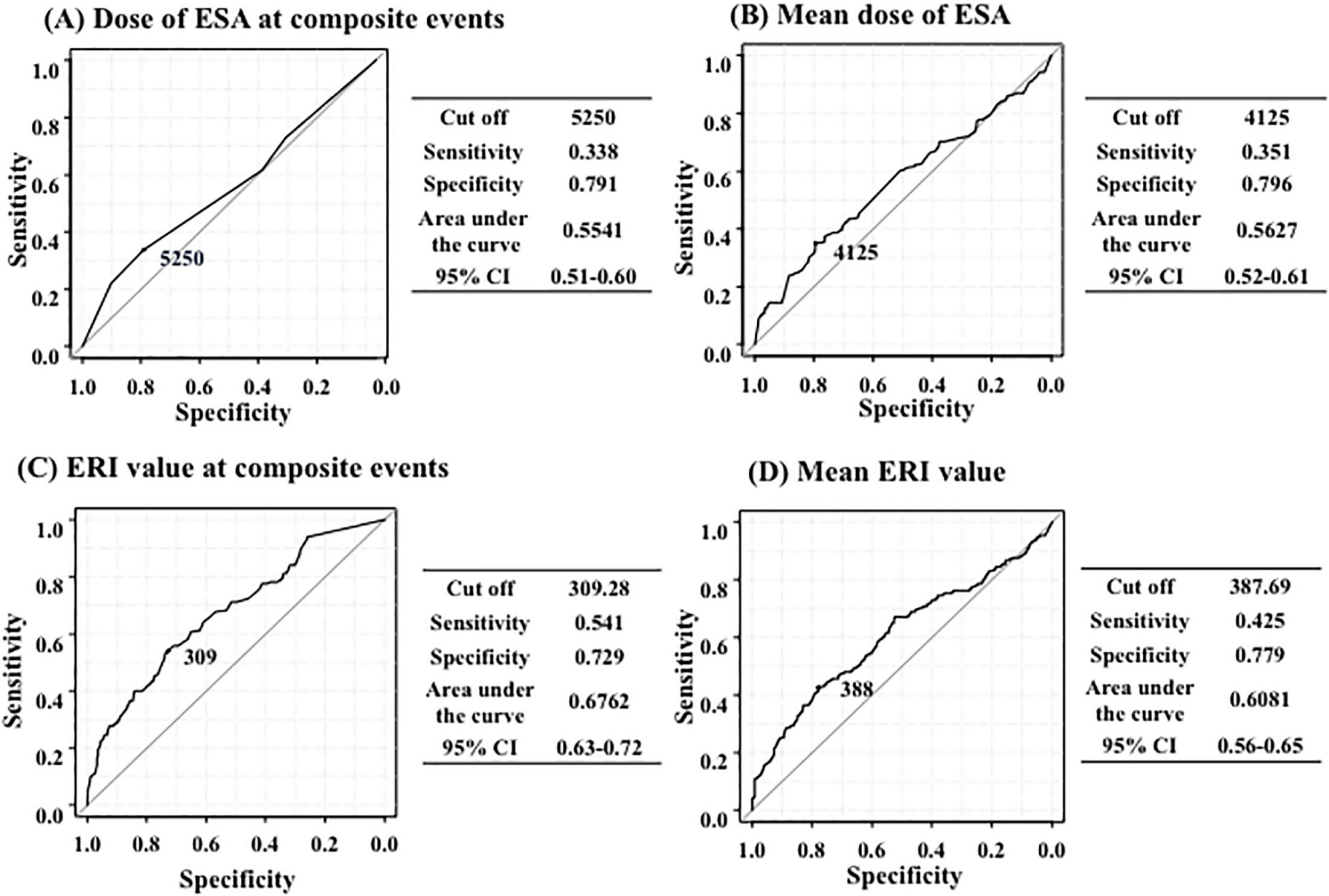
The cut-off values for the ESA dose and ERI for composite events. The cut-off values for the ESA dose (A) and ERI (C) at the time of the composite event were 5250 and 309.28 IU/week, respectively. The cut-off values for the mean ESA dose at the composite event (B) and mean ERI (D) until the composite event were 4,125 and 387.69 IU/week, respectively. According to the Hanley and McNeil method, the area under the curve for the ERI value at the time of the composite event was significantly higher than that of the ESA at the time of the composite event.

Using ROC analysis, the cut-off values for the ESA dose at the time of the composite events and the mean ESA dose in the patients without composite events during the observational period were 5,250 IU/week (sensitivity, 0.338; specificity, 0.7919) and 4,125 IU/week (sensitivity, 0.351; specificity, 0.796), respectively. The ERI cut-off at the time of the composite events and the mean ERI in the patients without composite events during observational period were 309.28 (sensitivity, 0.541; specificity, 0.7929) and 387.69 (sensitivity, 0.425; specificity, 0.779), respectively (Fig 4).

**Fig 5:**
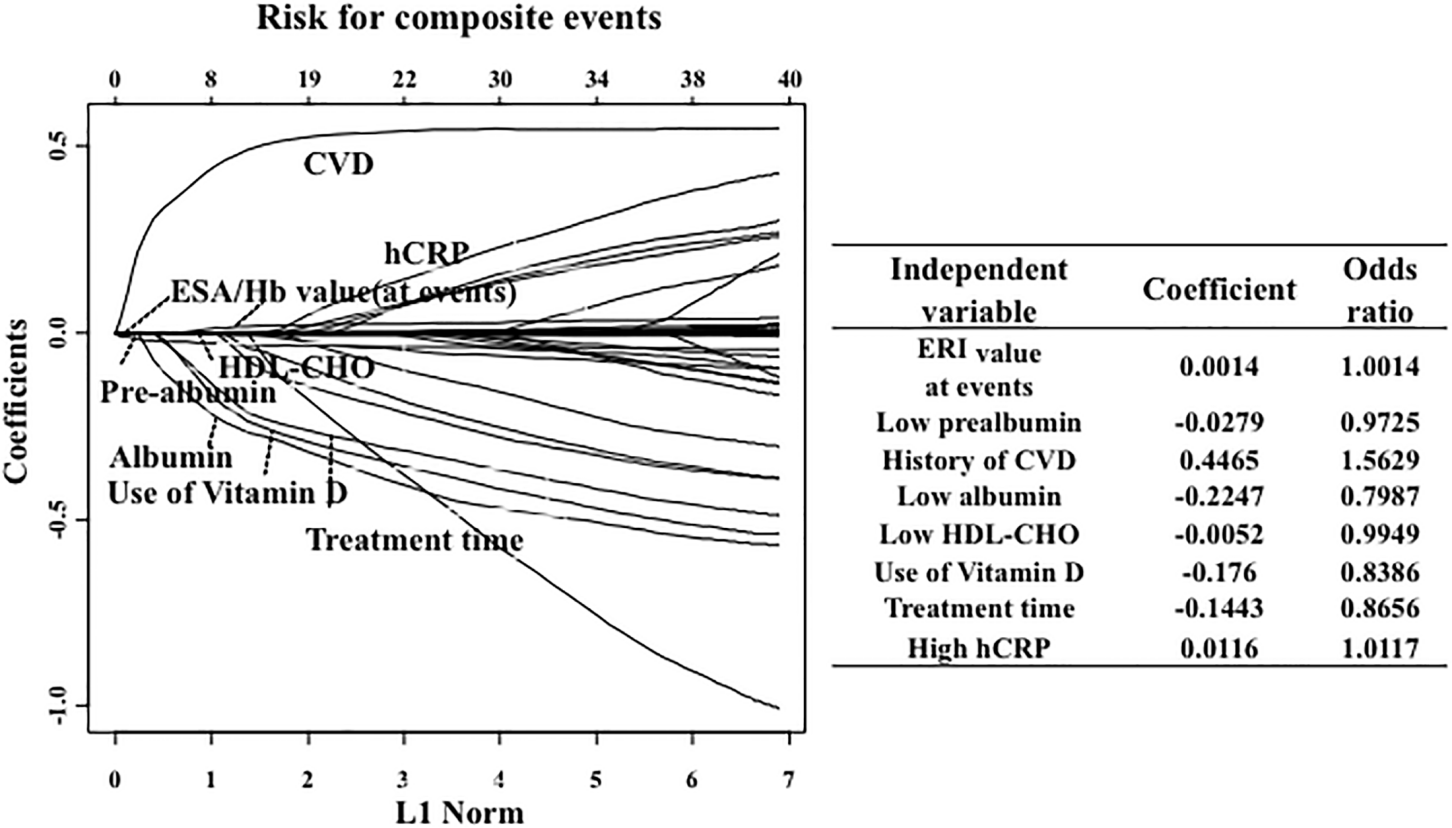
Factors associated with composite events. In the LASSO analysis, the ERI at the time of the composite events, low prealbumin, albumin and high density lipoprotein cholesterol (HDL-CHO) levels, a history of CVD, use of vitamin D at the time of treatment, and high hCRP levels were the predictors of composite events in maintenance hemodialysis patients.

When comparing the independent ROC curve data, it was observed that the area under the curve for ERI during composite events was significantly (p<0.001) higher than that of the ESA dose. In the LASSO regression analysis, a higher ERI during composite events (odds ratio, 1.0014), lower serum prealbumin levels (odds ratio, 0.9725), a history of CVD (odds ratio, 1.5629), lower serum albumin levels (odds ratio, 0.7987), lower serum HDL cholesterol levels (odds ratio, 0.9949), no vitamin D administration (odds ratio: 0.8386), a shorter treatment (dialysis) duration (odds ratio, 0.8656), and higher serum hCRP levels (odds ratio, 1.0017) were selected as significant predictors of composite events (Fig 5).

**Fig 6:**
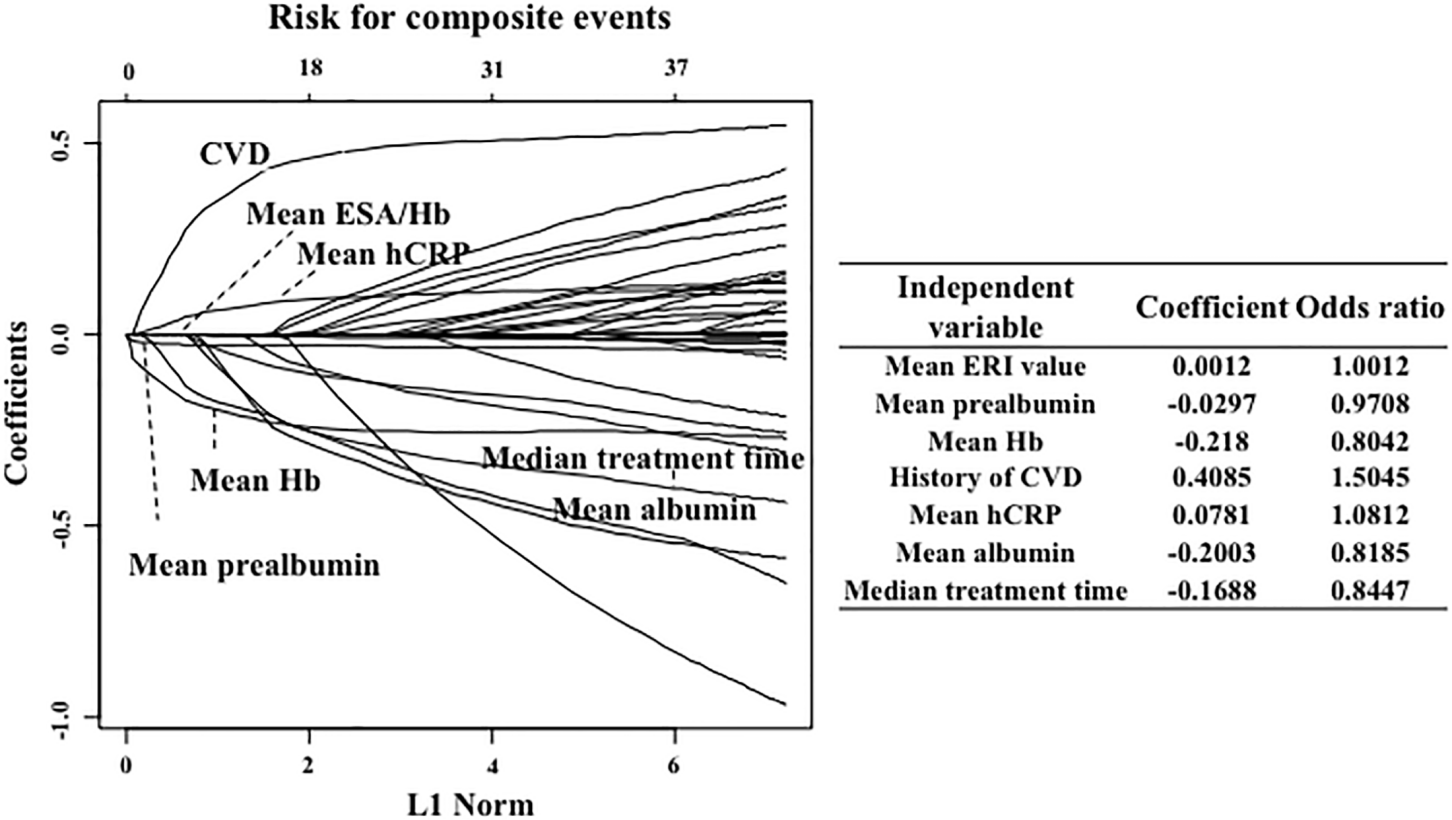
Correlation between mean or median levels of parameters and composite events. In the LASSO analysis, the mean ERI during the study period, lower mean prealbumin levels (odds ratio, 0.9708), lower mean Hb levels, history of CVD, higher mean hCRP levels, lower mean albumin levels, and shorter median treatment time were predictors of composite events in maintenance hemodialysis.

In addition, the mean ERI during the study period (odds ratio, 1.002), lower mean prealbumin (odds ratio, 0.9708), lower mean Hb levels (odds ratio, 0.8042), history of CVD (odds ratio, 1.5045), higher mean hCRP levels (odds ratio, 1.0812), lower mean albumin levels (odds ratio, 0.8185), and shorter median treatment time (odds ratio, 0.8447) were selected as significant predictors of composite events (Fig 6).

### Effects of ERI, ferritin level, and iron dose on composite events

**Fig 7:**
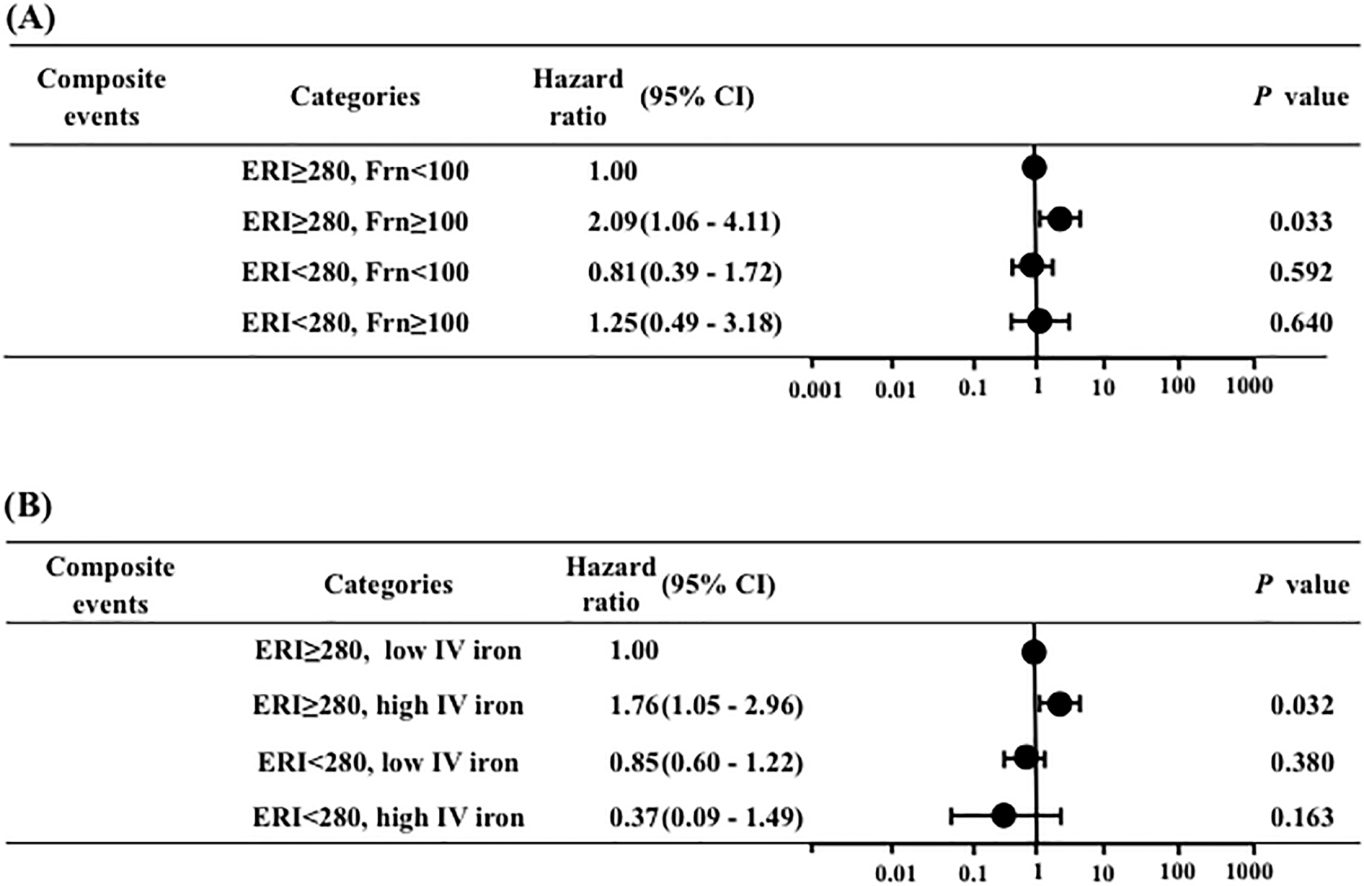
**A: Relationship among the combined categories of ERI, ferritin level and composite events**. The risk of composite events in patients with a high ERI (≥280) and a high ferritin (≥100 ng/mL) level was significantly greater (HR, 2.09; p = 0.033) than that of patients with a high ERI (≥280) and a low ferritin (<100 ng/mL) level. B: Relationship among the ERI value, intravenous iron dose and composite events. The risk of composite events in patients with a high ERI value (≥280) treated with a high dose of intravenous iron was significantly greater (HR: 1.76, p = 0.032) than that in patients with a high ERI value (≥280) treated with a low dose of intravenous iron. Frn: ferritin; low intravenous iron: treated with intravenous iron (<50 mg/week); high intravenous iron: treated with intravenous iron (≥50 mg/week).

In a time-dependent Cox hazard model, the risk for composite events in patients with a high ERI (≥280) and a high ferritin level (≥100 ng/mL) was significantly higher (HR: 2.09, p = 0.033) than that in the patients with a high ERI (≥280) and a low ferritin level (<100 ng/mL) (Fig 7A). When the patients were further subdivided into low-dose intravenous iron (<50 mg/week) and high-dose intravenous iron (≥50 mg/week) groups, the risk for composite events in patients with a high ERI (≥280) who were treated with a high dose (≥50 mg/week) of intravenous iron was significantly (HR, 1.76, p = 0.032) higher than that of patients with a high ERI (≥280) who were treated with a low dose (<50 mg/week) of intravenous iron (Fig 7B).

## Discussion

In this study, we determined that although patients with ESA hyporesponsiveness exhibit a high risk of composite events, iron storage and iron administration were not associated with a sustainable, improved responsiveness to ESA. Moreover, among patients with ESA hyporesponsiveness, patients with repleted iron stores or patients treated with higher doses of intravenous iron had a higher risk of composite events.

### Association between ESA or ERI and composite events

The Mann-Whitney U test indicated that a higher ESA dose and higher ERI were significantly associated with a greater risk of composite events. Furthermore, the LASSO analysis revealed that ERI was a predictor of composite events in MHD patients, as were low pre-albumin and HDL cholesterol levels, a history of CVD, no vitamin D administration, shorter treatment times, and high serum CRP levels. Interestingly, ERI was more closely associated with composite outcomes than other classical risk factors, such as low serum albumin and high CRP levels and a history of CVD.

Several studies have reported that ESA responsiveness (but not the ESA dose) is a major determinant of the occurrence of adverse events in MHD patients. Solomon S. D reported that patients with a poor response to ESA were at increased risk for the composite CVD end point (HR, 1.31) or death (HR, 1.41) compared with patients who had a better response (2). However, Bradbury reported that a larger increase in the ESA dose alone was not associated with increased mortality when the patients’ Hb levels remained under 9.5 g/dL after three months of being administered a large increased ESA dose [14]. In addition, it has been reported that ESA treatment is associated with increased thromboembolic events [15]. Furthermore, sub-analysis of the Correlation of Hemoglobin and Outcomes in Renal Insufficiency (CHIOR) trial revealed that a higher dose of epoetin-α (>10,095 units/week) was associated with an increased risk of cardiovascular events [16]. However, the mean dose of ESA in the present study was much lower (3,164 IU/week) than that of the sub-analysis in the CHIOR trial, which demonstrated the close relationship between a high ESA dose and cardiovascular events.

In this study, as observed in other studies, both higher ERI scores and higher ESA doses were significantly associated with the risk of composite events. However, the area under the ROC curve analysis of ERI for composite events was significantly higher than that of the ESA dose. Thus, we presumed that ESA responsiveness, not the ESA dose, might play an important role in the risk of composite events.

### Factors associated with ESA resistance

In the LASSO analysis, a high dose of intravenous iron administration, low serum calcium levels, female gender, low serum albumin levels, ACE-I/ARB use and low serum prealbumin levels were selected as significant predictors of a high ERI. These results mostly confirm the findings of previous studies [6–7].

The association between gender and ESA hyporesponsiveness has been previously reported [17]. The difference in ESA hyporesponsiveness between males and females has been explained by the difference in the release of iron from reticuloendothelial cells to the bone marrow for erythropoiesis [18–20]. In this study, the serum levels of albumin and prealbumin, which are markers of the nutritional state, were selected as significant predictors of ERI. The correlation between malnutrition and ESA hyporesponsiveness has been already established [6, 17]. ACE-I/ARB use has also been reported to be an independent predictor of the index of ESA resistance [7]. ACE-Is appear to cause anemia through a decreased production of red blood cells. Previous studies [21–22] have suggested that ACE-Is might impair erythropoiesis by suppressing either N-acetyl-seryl-aspartyl-lysyl-proline (AcSDKP)-mediated erythropoietin production or the bone marrow’s response to erythropoietin [23].

In the present study, we did not determine why low calcium was associated with higher ERI. Recently, the focus has been directed on vitamin D deficiency as a possible cause of ESA hyporesponsiveness. An inverse association between 25(OH)D3 levels and the EPO resistance index was observed in HD patients [24], and the administration of vitamin D or its analogs has been associated with improved anemia and/or reduced EPO requirements [25–26]. Furthermore, Bacchetta J et al. suggested the possibility that vitamin D regulates the expression of hepcidin in both in vivo and in vitro studies [27]. Although we did not evaluate the serum levels of vitamin D in this study, it is possible that low serum calcium levels could be caused by a vitamin D deficiency. If this hypothesis is correct, we could postulate that a vitamin D deficiency would result in low serum calcium levels and, thus, affect ESA responsiveness.

We found no significant correlation between hCRP or int-PTH and ERI, despite previous studies [6–7] demonstrating a relationship between inflammation or hyperparathyroidism and ESA responsiveness. In the current study, patients with severe inflammation or hyperparathyroidism were not included (the mean hCRP and int-PTH levels were 0.29±1.23 mg/dL and 148 ± 121 pg/mL, respectively), which may explain the discrepancy between this study and previous studies. Thus, we could not rule out the possibility that hCRP and int-PTH could be significant predictors of ERI.

Relationship between iron storage and ERI: In the cross-sectional analysis, we observed no significant difference in the Hb level and ESA dose among the four ferritin groups (<50, 50–100, 100–300, and ≥300 ng/mL). We observed a significant correlation between Hb and ferritin levels only in patients with ferritin levels below 50 ng/mL. Therefore, it was possible that iron administration in patients with ferritin levels below 50 ng/mL might contribute to the improvement of ESA responsiveness and anemia. Furthermore, serum ferritin and TSAT levels were not selected as significant predictors of the ESA resistance index. Similar to our results, the annual report of the Japan Society for Dialysis Therapy (JSDT) (2012) (http://www.jsdt.or.jp) showed that ESAI (ESA response index) scores in patients with low serum ferritin (<50 ng/mL) and high serum ferritin (>300 ng/mL) levels were elevated compared with those of the other ferritin groups (50–300 ng/mL). Moreover, there was no significant difference in ESAI score among the patients in the other three ferritin groups (50–99, 100–199, and 200–299 ng/mL).

A prospective, observational cohort study of 349 MHD patients in Japan also reported that patients with ESA hyporesponsiveness [low Hb (<10 g/dL) and a high ESA dose (>93.3 U/kg/week)] had a significantly higher risk of death. The authors also reported that the serum ferritin levels in patients with ESA hyporesponsiveness were significantly higher (159.6 vs. 78.7 ng/mL) than those in patients with a better response to ESA (high Hb (>10 g/dL) and low ESA dose (<93.3 U/kg/week)) [28]. From these results, we presumed that repleted iron stores do not necessarily lead to erythropoiesis or to improved ESA responsiveness in MHD patients.

### Relationship between iron administration and ERI

Although the ERI is a significant predictor of composite events in MHD patients, a high dose of intravenous iron administration was selected as an independent factor of ESA hyporesponsiveness using LASSO analysis. Previous studies have reported that even when MHD patients exhibited repleted iron storages, intravenous iron administration increased the Hb levels and reduced the ESA dose [8–9]. Although intravenous iron is highly effective in supplying iron to the bone marrow to accelerate erythropoiesis, higher doses of intravenous iron might result in increased ferritin levels or high iron storages in MHD patients. The effect on erythropoiesis from iron storage and exogenous iron administration may differ completely. Regarding the iron availability for erythropoiesis, it is essential to consider the role of hepcidin, a systemic regulator of iron metabolism. We and other authors have already demonstrated the close correlation between serum ferritin and hepcidin levels [29–30]. As iron storage (serum ferritin) increases, serum hepcidin levels also increase and degrade ferroportin, the only known iron export protein from cells. The decrease in ferroportin accelerates iron accumulation in reticuloendothelial cells and hampers the availability of iron for erythropoiesis. Conversely, exogenous iron enters the circulation and could be utilized for erythropoiesis for one-time only use, thereby escaping the effect of hepcidin. However, the reutilization of iron from senescent red blood cells should be affected by hepcidin. Thus, exogenous iron administration accelerates erythropoiesis, while excessive iron storage hampers it. Furthermore, it has been indicated that intravenous iron yields only small increases in Hb in exchange for the accumulation of relatively large amounts of iron [31].

### Iron storages or dose of intravenous iron on the adverse events

In this study, we demonstrated that among patients with high ERI levels, patients with high iron storages (≥100 ng/mL of serum ferritin) and patients treated with high doses (≥50 mg/week) of intravenous iron had significantly higher risks of composite events. Based on these results, we suspected that additional iron administration to patients with repleted iron storages might increase the risk of composite events in MHD patients, despite their high ERI values. The adverse effects of iron overload, oxidative stress and inflammation are chronic and insidious. The promotional use of intravenous iron to achieve arbitrary hemoglobin targets in patients who are hyporesponsive to ESA or as an ESA-sparing strategy might have adverse consequences [32]. From our previous reports regarding the TRAP study, high (>100 ng/mL) levels or upward/high-amplitude (<100 vs. ≥100 ng/mL) serum ferritin fluctuations and high intravenous iron doses were associated with a high risk of death and/or adverse events in MHD patients [11]. In the present study, we also demonstrated that patients with a high ERI (≥280) who were treated with a high dose of intravenous iron (≥50 mg/week) had a significantly greater risk of composite events than patients with a high ERI who were treated with a low dose of intravenous iron (<50 mg/week). Furthermore, ESA hyporesponsiveness is associated with generalized illness, such as infection, and malnutrition. Administering a high dose of intravenous iron to these fragile patients might cause infection, CVD, or premature death. From these results, we presumed that the reduction of the ESA dose by a high dose of intravenous iron in patients with repleted iron stores is not associated with improved ESA responsiveness and better survival. Further investigation could help clarify the relationship between appropriate iron storage and iron administration in the treatment of renal anemia.

### Limitations

1) The sample size of this study was too small to determine the correlation between ERI and each event or survival. Therefore, we analyzed the correlation between ERI and the composite events. 2) The sensitivity of the cut-off values (dose of ESA at composite events, dose of ERI at composite events, mean dose of ESA, and mean ERI value) for composite events by the ROC analysis shown in Fig 4 were low (0.338, 0.541, 0.351, and 0.425, respectively). Therefore, it is difficult to determine the significant cut-off value for the dose of ERI for composite events due to the existence of false positives.

In conclusion, Hb levels were associated with serum ferritin levels in patients with <50 ng/mL ferritin, but not in patients with ≥50 ng/mL ferritin. Although patients with ESA hyporesponsiveness had a greater risk of composite events, iron storage, as indicated by serum ferritin levels, was unrelated to sustained improvements in ESA responsiveness. From these results, we concluded that repleted iron storage does not necessarily improve ESA responsiveness in MHD patients, although intravenous iron administration might transiently affect ESA responsiveness.
